# Dynamic Mechanical Properties of a Novel Wire-Mesh Composite Elastic Porous Metallic Material Under Low-Frequency Excitation

**DOI:** 10.3390/ma19214680

**Published:** 2026-11-03

**Authors:** Ying Han, Qing Tian, Huijian Wang, Yiwan Wu

**Affiliations:** 1 China National Nuclear Corporation Overseas Ltd., Beijing 100044, China; 2 Engineering Research Center for Metal Rubber, School of Mechanical Engineering and Automation, Fuzhou University, Fuzhou 350108, China

**Keywords:** metal rubber, wire-mesh composite, dynamic mechanical properties, low-frequency damping, loss factor, vibration isolation

## Abstract

**Highlights:**

**What are the main findings?**

- Dynamic stiffness and energy dissipation of WM-EPMM increase with excitation amplitude and material density.
- In the 1–5 Hz range, higher frequency slightly decreases stiffness but increases the loss factor, with constant dissipated energy.
- The mass ratio of crimped mesh and its crimping angle reduce all three dynamic performance metrics.
- Mechanisms are linked to a transition from line to point contact and the stability of equivalent inclined struts.

**What are the implications of the main findings?**

- The findings provide a strategy for tailoring low-frequency dynamic performance of metal rubber composites via architectural design.
- The findings offer data and insights for designing customizable dampers for low-frequency vibration control.
- The composite structure expands the design space for elastic porous metallic materials in targeted applications.

**Abstract:**

Elastic Porous Metallic Materials (EPMMs), also known as metal rubber, are used for damping and vibration isolation. In this study, a Wire-Mesh Composite Elastic Porous Metallic Material (WM-EPMM) is developed by integrating crimped metal mesh with conventional metal spiral coils to enable tailored dynamic performance. The dynamic mechanical properties of WM-EPMM, including average dynamic stiffness, loss factor, and dissipated energy per cycle, are experimentally investigated under sinusoidal displacement excitation. The results show that increasing excitation amplitude and material density leads to higher dynamic stiffness and energy dissipation, while the loss factor decreases moderately. Within the tested low-frequency range of 1–5 Hz, a higher excitation frequency slightly reduces stiffness and increases the loss factor, whereas the dissipated energy per cycle remains nearly unchanged. Furthermore, increasing the mass ratio of the crimped mesh or its crimping angle negatively correlates with all three dynamic performance metrics. These observations are interpreted through changes in internal contact states, specifically the transition from line to point contact, and a proposed equivalent inclined strut model for the crimped geometry. The findings provide experimental data and mechanistic insights that may inform the design of WM-EPMM components with adjustable dynamic properties for low-frequency vibration control applications.

## 1. Introduction

The mitigation of detrimental low-frequency vibrations and shocks is of critical importance across numerous engineering fields, such as precision machinery, vehicle suspension, and aerospace systems [1,2,3,4]. As a representative class of porous damping materials, Elastic Porous Metallic Materials (EPMMs), or metal rubber, have attracted considerable attention due to their unique combination of metallic durability, such as corrosion resistance and thermal stability, and rubber-like elasticity and damping capacity [5,6]. Conventional EPMM is typically fabricated by coiling, stretching, and cold-pressing metal wires into spiral coils, forming an entangled network wherein energy dissipation occurs primarily through dry friction between contacting wire segments [7,8].

To tailor mechanical performance for diverse applications, researchers have explored composite EPMM architectures. Strategies include employing wires with non-circular cross-sections [9], integrating woven wire meshes [10], and combining different materials or architectures [11,12,13,14,15,16,17,18,19,20]. These composite approaches include lead–stainless steel [11], beryllium bronze alloy [12], copper–steel wire [13], polyurethane interpenetrating phases [14], silicone rubber [15], metallic wire mesh structures [16], multi-strand wires [17], laminated metal rubber–silicone rubber structures [18], corrugated metal rubber composites [19], and tensegrity metamaterials [20]. Among these, the incorporation of metal mesh into the EPMM matrix offers a potential route for modulating stiffness and damping by altering internal contact mechanisms.

However, existing studies on mesh-modified EPMM have primarily focused on quasi-static or dynamic compressive properties of homogeneous coil-based EPMM under simple loading conditions [21,22,23,24,25,26,27,28,29,30]. These studies include dynamic compressive behavior of soft magnetic EMWM [21] and low-velocity impact constitutive models [22], sandwich metal rubber structures [23,26,27], numerical modeling of entangled single-wire metal rubber [24], quasi-static and impact behavior [25], metal-rubber dynamic vibration absorbers [28], and fatigue properties of multi-strand and entangled wire materials [29,30]. Systematic investigations into the dynamic mechanical behavior of wire-mesh composites remain relatively limited, especially for those incorporating pre-crimped meshes with controlled geometric parameters.

Moreover, EPMM is often used for low-frequency vibration isolation, such as in the 1–5 Hz range typical of machinery start-up, vehicle suspension, and structural base isolation. However, the quantitative influence of key manufacturing parameters, including the mass ratio of crimped mesh and its crimping angle, on dynamic stiffness, loss factor, and dissipated energy within this frequency band has not been fully established. In particular, it remains to be clarified how the transition from line contacts in coils to point contacts in mesh, as well as the orientation of crimped struts, affects low-frequency damping mechanisms.

It is also worth noting that the proposed WM-EPMM shares certain functional similarities with conventional metal foams, which are widely used for energy absorption and damping [31,32,33,34]. Metal foams typically exhibit brittle collapse or plateau stress behavior under compression, with limited recoverable deformation. In contrast, WM-EPMM shows recoverable elastic deformation up to relatively large strains, making it suitable for repeated cyclic loading. Additionally, WM-EPMM provides tunable stiffness and damping through architectural parameters, such as mesh mass ratio and crimping angle, whereas metal foam properties are primarily governed by relative density and cell morphology. However, WM-EPMM may have lower specific stiffness compared to some metal foams at equivalent density, and its manufacturing complexity is higher due to the crimping and assembly steps. A detailed comparison with consolidated metal foams, particularly regarding compressive behavior, is provided in Section 4.3 [31].

To address these gaps, the present work develops a Wire-Mesh Composite Elastic Porous Metallic Material (WM-EPMM) fabricated by combining pre-crimped metal mesh with conventional metal spiral coils. This study experimentally investigates the dynamic mechanical properties of the WM-EPMM under low-frequency harmonic loading in the 1–5 Hz range, a regime in which dry friction rather than rate sensitivity is expected to dominate energy dissipation. The effects of manufacturing parameters, including density (ρ), mass ratio of crimped mesh to total mass (*C*), and crimping angle (α), as well as excitation conditions, such as frequency (f) and amplitude (A), on the average dynamic stiffness (k), loss factor (η), and dissipated energy per cycle (ΔW) are systematically evaluated. The observed trends are interpreted through microstructural contact analysis, including a proposed equivalent inclined strut model for the crimped mesh. The results are intended to provide experimental data and mechanistic insights that may support the design of WM-EPMM components with adjustable dynamic characteristics for low-frequency vibration isolation applications building on the dry friction damping mechanism of metal rubber [35].

## 2. Materials and Methods

### 2.1. Specimen Preparation

**Figure 1:**
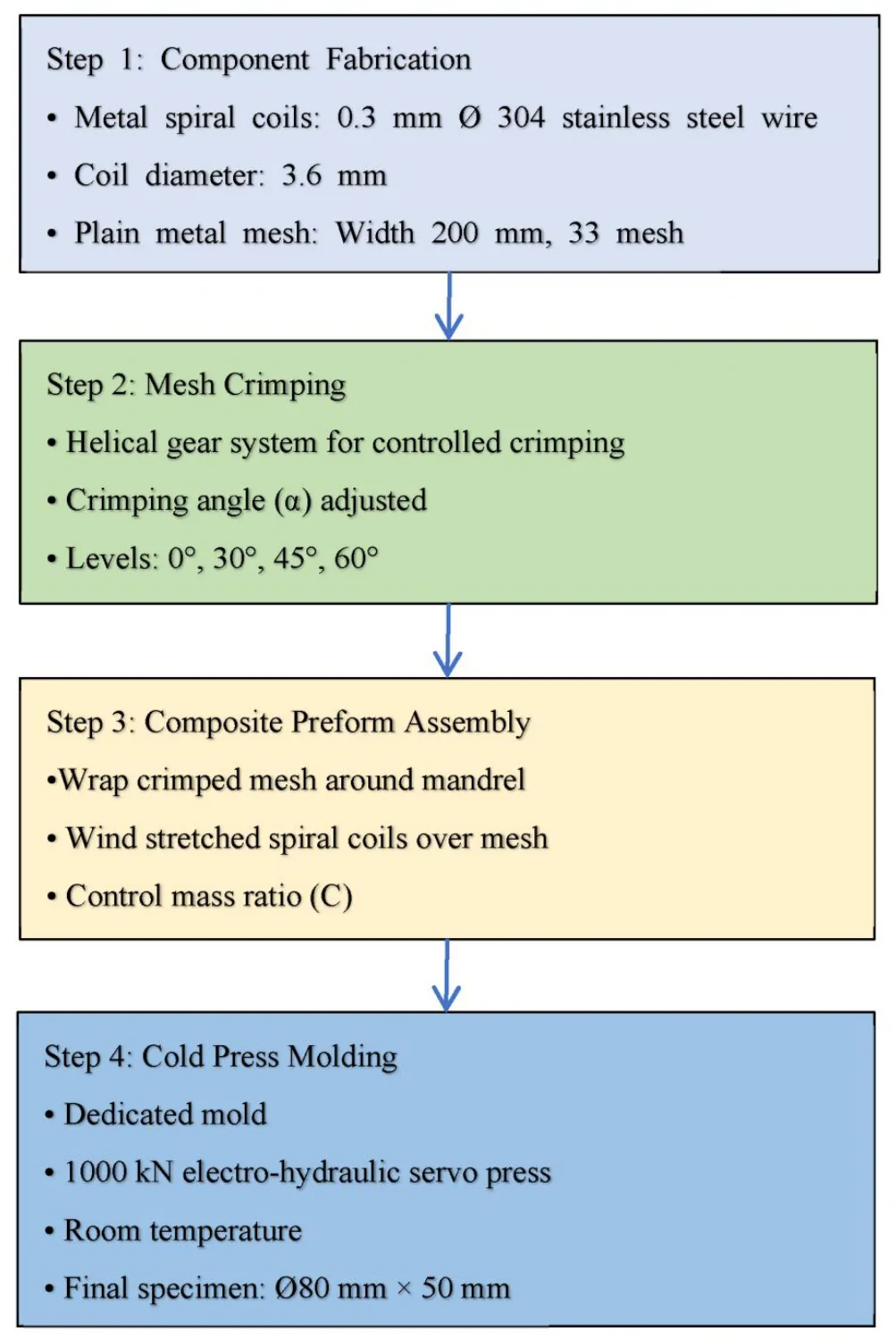
Schematic of the WM-EPMM fabrication process.

WM-EPMM specimens were manufactured using 0.3 mm diameter 304 stainless steel wires (0Cr18Ni9). As shown in Figure 1, the preparation process comprised four principal stages:

1. Component fabrication: Metal spiral coils were wound using an automatic wire-winding machine (Xiamen Zhihui Robot Co., Ltd., Xiamen, China), with a coil diameter of 3.6 mm. Plain metal mesh with a width of 200 mm and a mesh count of 33 per inch was woven on a circular weaving machine (Tianjin Tianyin Tengxiang Technology Co., Ltd., Tianjin, China).
2. Mesh crimping: The plain mesh was fed through a pair of helical cylindrical gears to create a regular crimped pattern. The crimping angle α, defined as the angle between the crimp ridge and the horizontal plane, was controlled to 0°, 30°, 45°, and 60°.
3. Composite preform assembly: Pre-crimped mesh and stretched spiral coils were weighed to achieve the target mass ratio C, which is the mass of crimped mesh divided by total mass. The mesh was first wrapped around a mandrel, followed by winding the coils over it to form a cylindrical preform.
4. Cold press molding: The preform was placed in a dedicated mold and compressed using a 1000 kN electro-hydraulic servo press (Tianjin Huidian Electromechanical Equipment Co., Ltd., Tianjin, China) at ambient temperature to produce the final cylindrical specimen with a diameter of 80 mm and a height of 50 mm.

**Figure 2:**
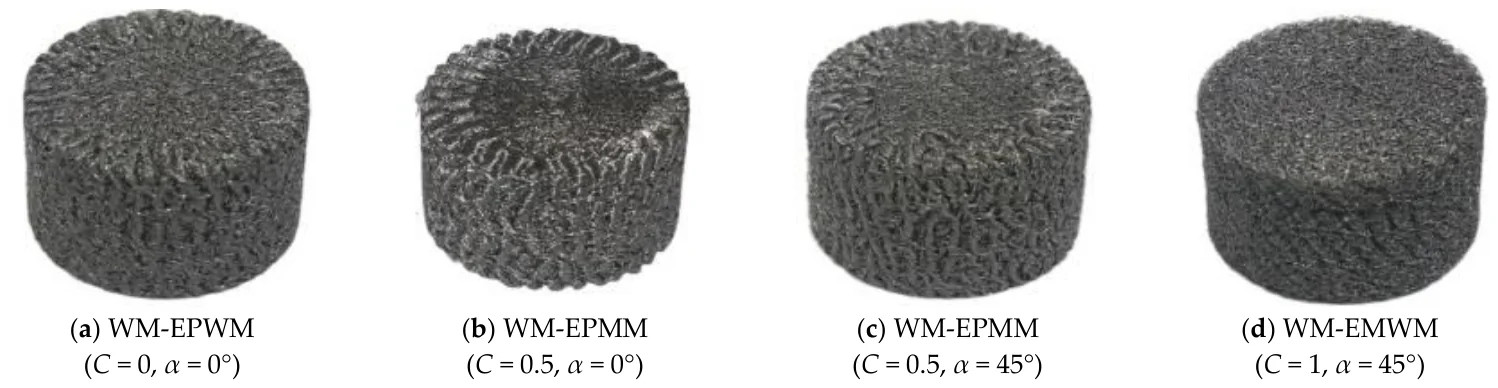
Photographs of representative specimens.

**Figure 3:**
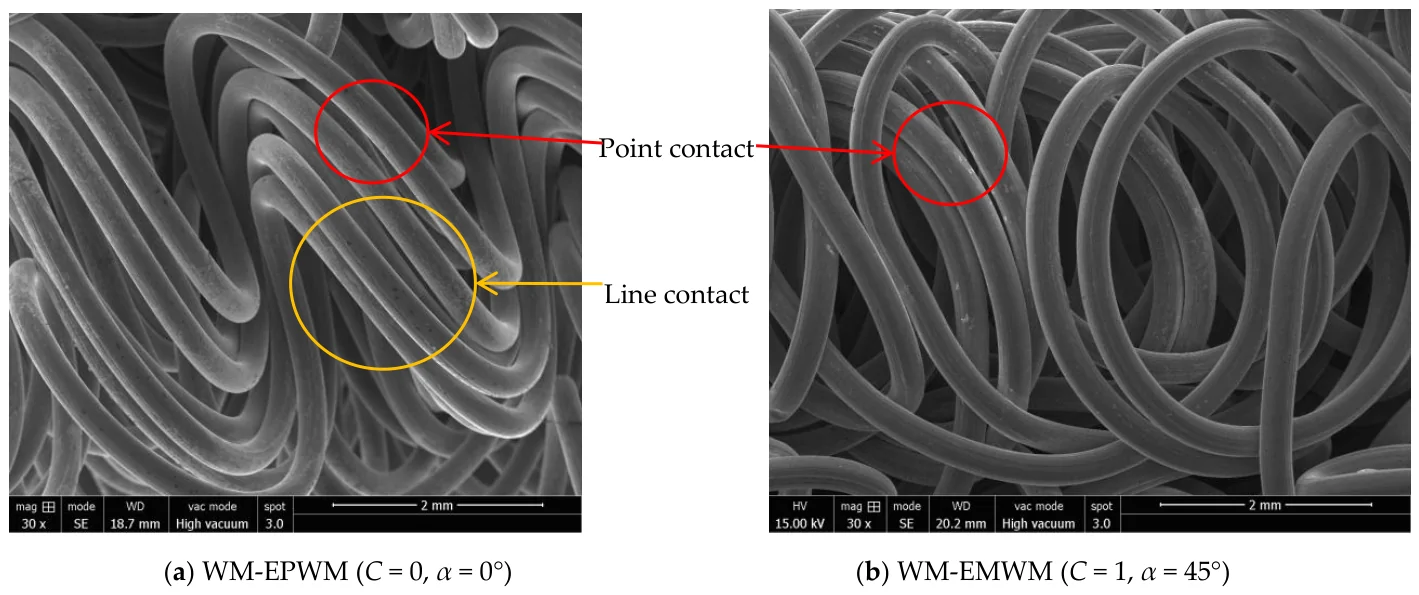
Microstructure of representative specimens (line contact vs. point contact).

For comparison, baseline specimens of traditional coil-based EPMM with C = 0 and uncompressed mesh composite with α = 0° were also prepared with identical geometries and densities. The apparent densities of the specimens ranged from 1.5 to 2.25 g·cm^−3^. Representative photographs and the distinctive micro-contact morphologies, namely line contact in coils and point contact in mesh, are compared in Figure 2 and Figure 3.

### 2.2. Dynamic Mechanical Testing

Dynamic tests were conducted on an SDS-200 servo-hydraulic testing machine (Changchun Mechanical Science Research Institute Co., Ltd., Changchun, China). The specimen was positioned between two parallel platens, and a pre-compression of 4 mm was applied to ensure stable contact. This pre-compression also served to eliminate initial loose contacts within the specimen. Sinusoidal displacement excitations *y*(*t*) = *A*sin(2*πft*) were then imposed, and the resulting force was recorded at a sampling frequency of 5000 Hz. The force signals were recorded directly from the testing machine, and no additional filtering was applied before hysteresis integration.

Each test condition was performed on a single specimen. To ensure stable and representative data, each specimen was subjected to pre-cycling until stable hysteresis loops were obtained, which typically occurred after five cycles. The data from one stable cycle were then used for analysis. The limitations of single-specimen testing are acknowledged in the Conclusions. A single-variable approach was adopted. One parameter was varied at a time according to Table 1, while others remained at standard values. For the excitation tests in Table 2 and Table 3, the standard test conditions were *f* = 1 Hz, *A* = 2 mm, *ρ* = 2.0 g·cm^−3^, *C* = 0.5, and *α* = 45°. For manufacturing parameter tests, *ρ* varies in Table 4, while the standard density for Table 5 is *ρ* = 1.75 g·cm^−3^. The excitation frequency was varied within the 1–5 Hz range to characterize the low-frequency dynamic response.

Tests were conducted from lowest to highest amplitude and frequency to minimize conditioning effects. Each specimen was used for only one test condition to avoid cumulative damage, and fresh specimens were prepared for each condition.

### 2.3. Dynamic Performance Characterization

**Figure 4:**
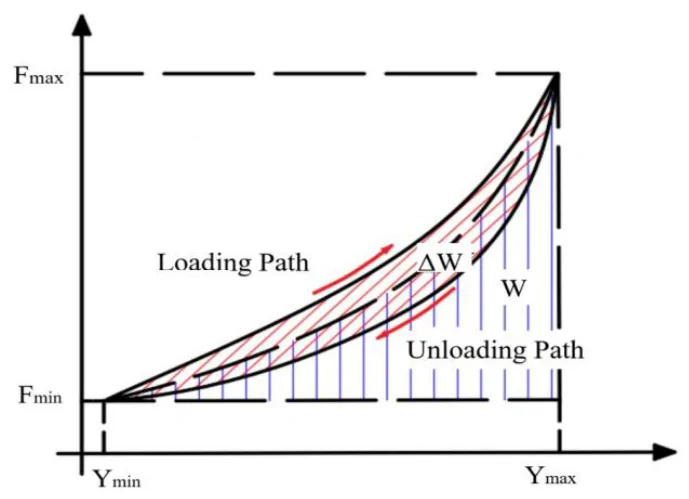
Typical hysteresis loop of EPMM.(Note: the red hatched area represents the energy dissipated per cycle, and the blue vertically striped area represents the elastic potential energy stored during loading.).

From the acquired force–displacement hysteresis loops (Figure 4), the following metrics were derived:

① Average dynamic stiffness *k*:
  $$k=\frac{F_{m\mathrm{ax}}-F_{m\mathrm{in}}}{y_{m\mathrm{ax}}-y_{m\mathrm{in}}}$$
② Dissipated energy per cycle Δ*W*, which is the area enclosed by the hysteresis loop and computed via discrete integration.
③ Loss factor *η*:
  $$\eta =\frac{\Delta W}{\mathrm{\pi U}}$$
  where *U* denotes the maximum elastic strain energy stored per cycle. In this study, *U* is calculated as the area under the loading curve from *y*_min_ to *y*_max_ minus half of the dissipated energy:
  $$U=\int_{y_{m\mathrm{in}}}^{y_{m\mathrm{ax}}}F_{l\mathrm{oading}}\;\mathrm{dy}-\frac{\Delta W}{2}$$

This definition accounts for the pre-compression offset by using the actual loading curve rather than assuming a symmetric loop [7,22].

## 3. Results

### 3.1. Effects of Excitation Conditions

#### 3.1.1. Excitation Amplitude

**Figure 5:**
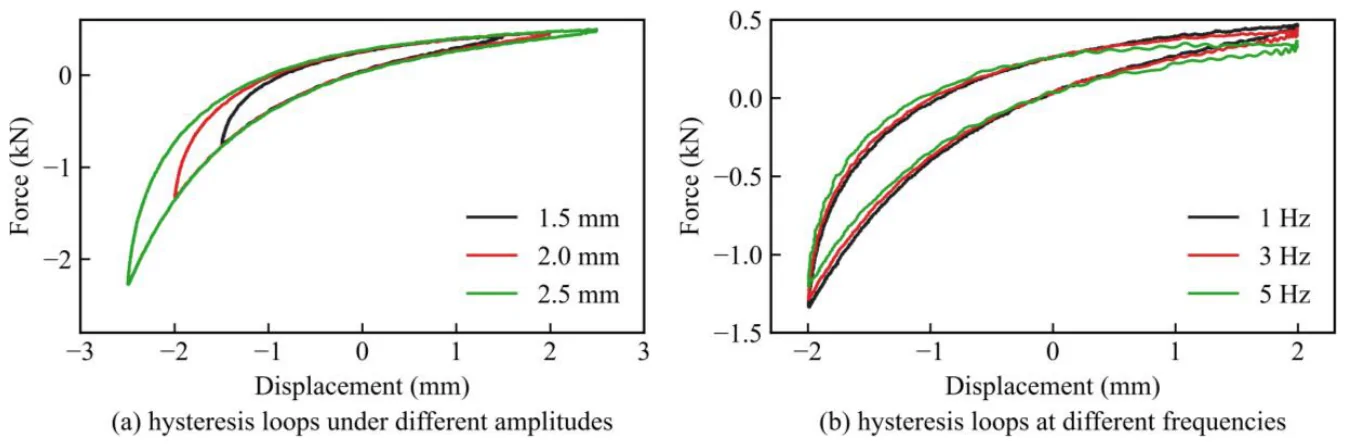
Dynamic hysteresis loops under varying excitation conditions.

Figure 5a presents hysteresis loops under different amplitudes. Both loop area and slope increase with *A*. As summarized in Table 2, *k* and Δ*W* increase by approximately 38% and 142%, respectively, when A rises from 1.5 to 2.5 mm, whereas *η* decreases by about 10%.

Furthermore, it should be noted that the applied load in this study was limited to approximately 2.5 kN. At higher loads, the material is expected to undergo significant densification, leading to a rapid increase in stiffness. Once the local contact stresses exceed the yield strength of the 304 stainless steel wires, plastic deformation will occur at the contact points, causing permanent structural changes and a decrease in recoverable elastic strain. Consequently, the energy dissipation mechanism would shift from pure dry friction to a combination of friction and plastic yielding.

#### 3.1.2. Excitation Frequency

Figure 5b displays hysteresis loops at different frequencies within the 1–5 Hz range. While Δ*W* remains nearly constant at approximately 0.927 J, the loop shape shifts slightly downward, and its slope diminishes as *f* increases. Consequently, *k* decreases by about 13%, whereas *η* increases by approximately 31% (Table 3).

#### 3.1.3. Consideration of the Tested Frequency Range

The dynamic tests were conducted within the 1–5 Hz range to specifically target low-frequency vibration scenarios, such as those arising from machinery start-up, vehicular suspension, or structural base isolation. In this quasi-static and low-frequency regime, the dissipated energy Δ*W* remained largely frequency-independent (Table 3), confirming that the dominant energy dissipation mechanism is amplitude-dependent dry friction, consistent with the fundamental behavior of metal rubber materials [7,8]. This focus allows for a clear investigation of how architectural parameters affect performance in this operationally relevant frequency band without the confounding effects of strain-rate-sensitive mechanisms that may become significant at much higher frequencies above 50 Hz. At higher frequencies, additional mechanisms, such as viscoelastic effects, inertial effects, and possible self-heating may become significant, and a possible transition from amplitude-governed to rate-governed dissipation could occur. Future work to characterize the frequency-dependent behavior across a broader spectrum would be helpful to further map the dynamic performance of WM-EPMM.

### 3.2. Effects of Manufacturing Parameters

**Figure 6:**
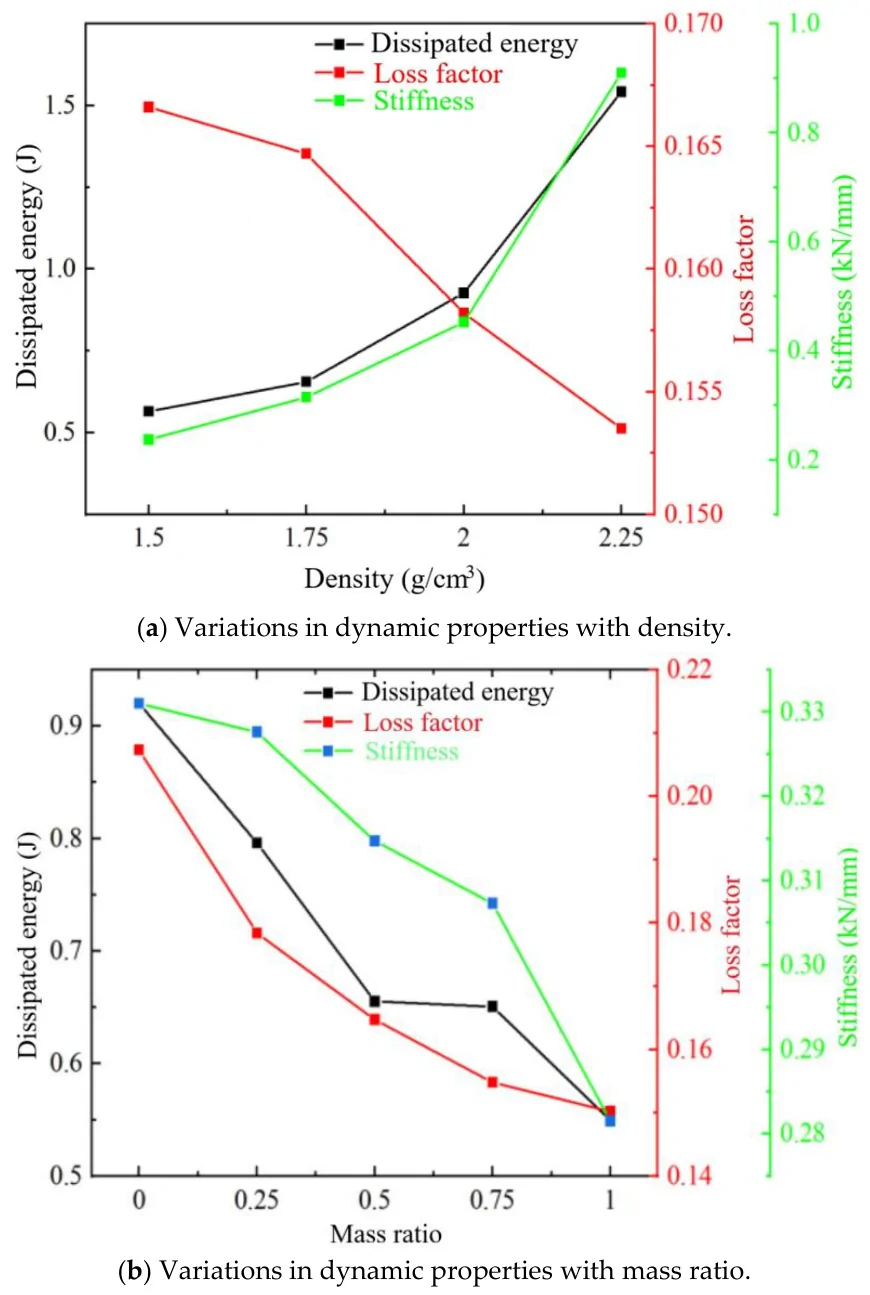

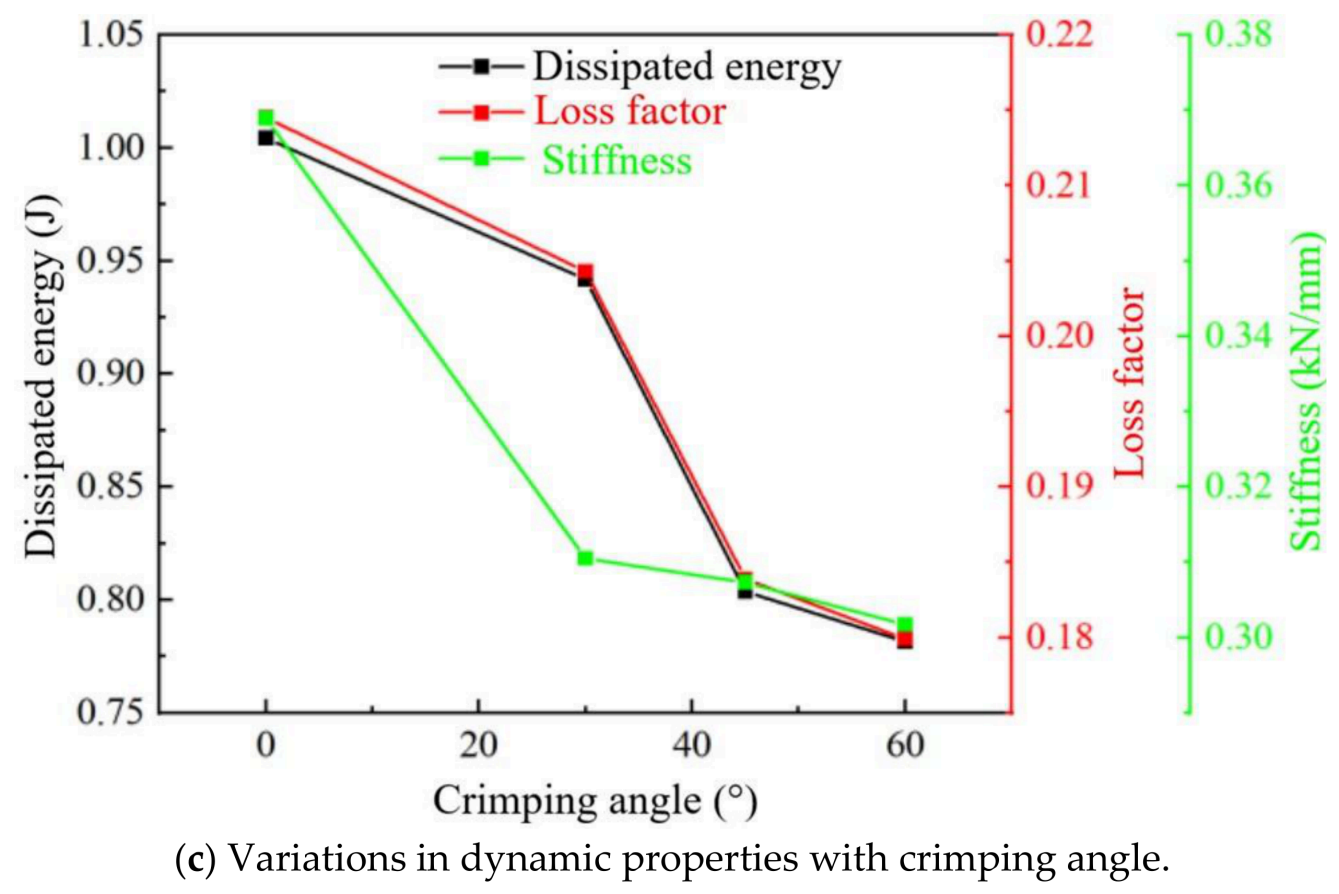
Variations in dynamic properties with key manufacturing parameters.

The comprehensive effects of density (*ρ*), mass ratio (*C*), and crimping angle (*α*) on *k*, *η*, and Δ*W* are quantified in Figure 6.

#### 3.2.1. Density

As shown in Figure 6a and Table 4, increasing density from 1.5 to 2.25 g·cm^−3^ leads to increases in both *k* (~284%) and Δ*W* (~173%), while *η* decreases gradually by approximately 8%.

#### 3.2.2. Mass Ratio of Crimped Mesh (C)

Figure 6b shows a negative correlation between *C* and all three performance metrics. Compared to pure coil EPMM with *C* = 0, WM-EPMM with *C* = 1 exhibits reductions of 15% in *k*, 40% in Δ*W*, and 28% in *η*.

#### 3.2.3. Crimping Angle (α)

The crimping angle influences the dynamic response, as shown in Figure 6c and Table 5. Increasing *α* from 0° to 60° reduces *k* by 18%, Δ*W* by 29%, and *η* by 16%.

## 4. Discussion

### 4.1. Mechanisms of Amplitude and Density Effects

The observed trend is attributed to the evolution of internal contact states. Larger deformations compress the porous structure, reducing pore size and causing initially non-contacting or sliding wire segments to enter tightly packed bearing states, as described by the cell group energy dissipation mechanism [35]. The increase in effective contact points and the shortening of micro-beam lengths between contacts lead to a higher macroscopic stiffness. Concurrently, the greater sliding distance between contacts lead to a higher macroscopic stiffness. The greater sliding distance between wires increases frictional energy dissipation, which corresponds to a higher Δ*W*. However, because the stored elastic energy *U* grows more rapidly than Δ*W*, the ratio *η* shows a moderate decline. Higher density corresponds to more wire material per unit volume, resulting in a greater number of potential contact points and shorter, stiffer micro-beams within the entangled network. This enhances both load-bearing capacity and frictional dissipation [15,25], consistent with previous observations on density effects in EMWM-silicone rubber composites [15] and quasi-static/impact behavior [25].

### 4.2. Mechanisms of Frequency Effects

The near-constant Δ*W* suggests that, within this low-frequency range, energy dissipation per cycle is predominantly amplitude-governed rather than rate-sensitive, which is characteristic of dry friction damping. The reduction in *k* may be due to incomplete response of internal frictional contacts at higher frequencies, resulting in a lag and lower apparent stiffness. The downward shift of the loop reduces the stored elastic energy *U*. Since Δ*W* remains stable, a smaller *U* yields a higher calculated loss factor *η*. This frequency-insensitive dissipation further supports the dominance of dry friction in the tested range, though behavior at higher frequencies may involve additional rate-dependent mechanisms that warrant further investigation [28,29,30,36], as suggested by studies on metal–rubber dynamic vibration absorbers [28], fatigue under cyclic dynamic loads [29,30], and strain-rate sensitivity of entangled wire materials [36].

### 4.3. Comparison with Metal Foams

The proposed WM-EPMM shares certain functional similarities with conventional metal foams, which are widely used for energy absorption and damping [31,32,33,34]. To provide a direct comparison regarding compressive behavior, we specifically refer to the recent work on closed-cell Al alloy foams by Giudice et al. [35]. The compressive stress–strain curve of conventional closed-cell metal foams typically exhibits three distinct regimes: a linear elastic regime, a long plastic collapse regime characterized by a nearly constant plateau stress, and a final densification regime where the stress rises rapidly due to cell wall crushing and pore compaction. This plateau stress behavior is primarily determined by the morphological characteristics of the cellular structure (e.g., cell size, circularity, and wall thickness) and their evolution during the collapse process.

In contrast, the WM-EPMM investigated in this study exhibits a fundamentally different compressive behavior. Compared with consolidated metal foams [31,35], the WM-EPMM exhibited recoverable elastic deformation up to relatively large strains, making it suitable for repeated cyclic loading. As shown in our quasi-static and dynamic tests, the WM-EPMM curves do not present a traditional plateau stress. Instead, they display a continuous stiffening trend due to the progressive transition from line to point contact and the densification of the entangled wire network. Furthermore, while metal foams rely on the plastic hinging or brittle crushing of the solid cell walls for energy dissipation, the WM-EPMM dissipates energy primarily through the dry friction and sliding between metallic wires.

The WM-EPMM also provides tunable stiffness and damping through architectural parameters, while metal foam properties are primarily governed by relative density and cell morphology. However, the WM-EPMM may have lower specific stiffness at equivalent density, and its manufacturing complexity is higher. These trade-offs should be considered when selecting materials for specific vibration isolation applications.

### 4.4. Mechanisms of Mass Ratio and Crimping Angle

The primary mechanism for the mass ratio lies in the difference in internal contact geometry. As shown in Figure 3, in coil-based regions, contact between adjacent spiral turns is predominantly line contact, whereas in crimped mesh regions, contact between wires is essentially point contact [10], as observed in composite elastic–porous metal materials. This contact mode transition may contribute to the observed trends, similar to the topological analysis of entangled single-wire metal rubber [24]. Line contact offers a larger effective sliding area and can be modeled as multiple parallel micro-springs, contributing to higher stiffness and friction. In contrast, point contact provides less constrained sliding and behaves more like slender bending beams with lower stiffness. As the proportion of mesh (point contact) increases, the overall structure becomes more compliant and exhibits reduced energy dissipation capacity.

**Figure 7:**
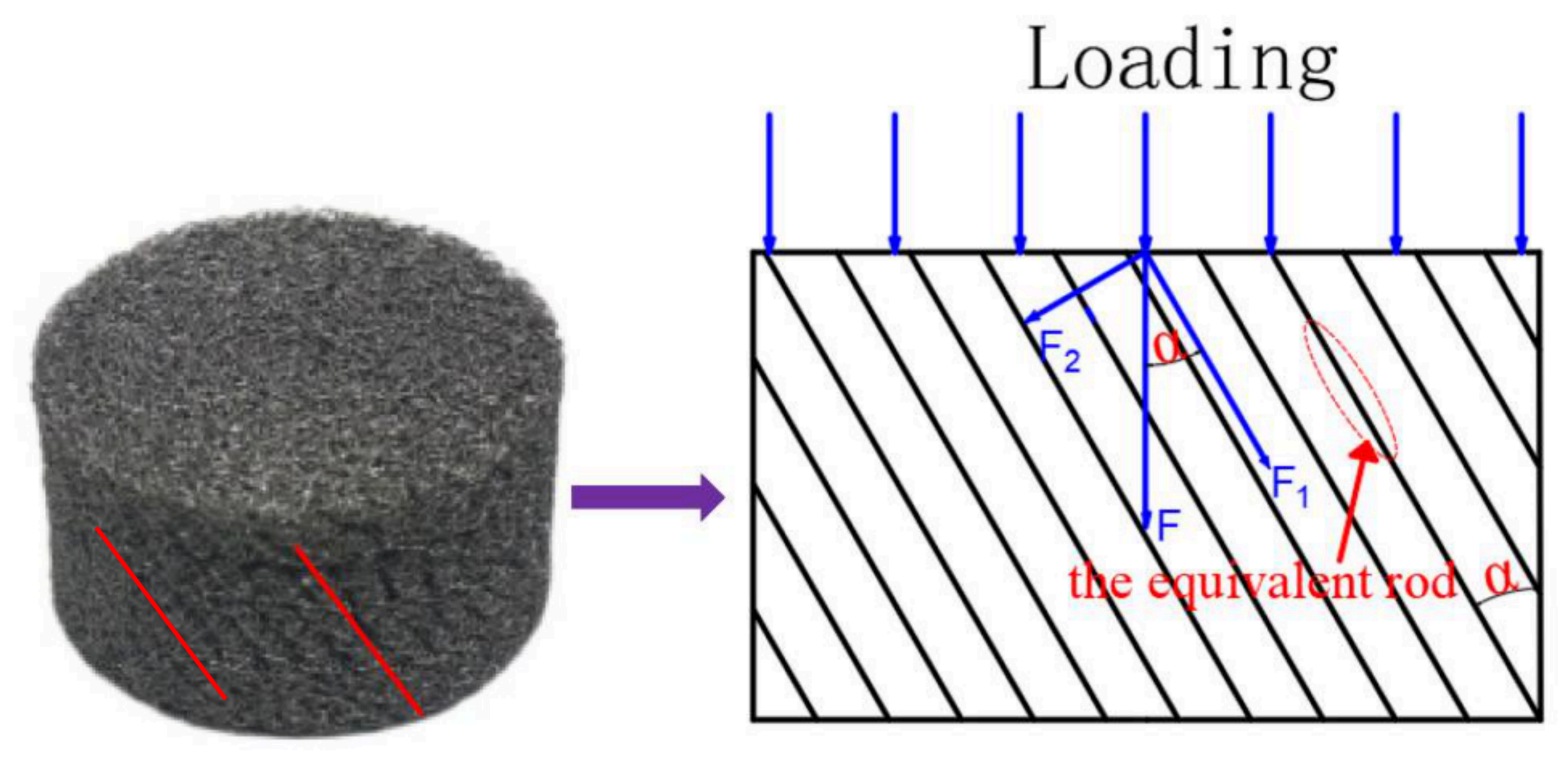
Schematic diagram of force decomposition of EPMM with different embossing.

For the crimping angle, the crimped mesh can be conceptually simplified as a series of equivalent inclined rods within the composite, as shown in Figure 7. Under axial compression, the applied force *F* on WM-EPMM can be resolved into a component along the strut, denoted *F*_1_, and one perpendicular to it, denoted *F*_2_. With increasing α, *F*_1_ decreases, while *F*_2_ increases, which promotes strut rotation and buckling. This tends to reduce the structural stability under load, leading to lower effective stiffness. Reduced stiffness and constrained deformation also limit the sliding motion necessary for friction, thereby lowering Δ*W* and *η*. It should be noted that this equivalent inclined strut model is a conceptual and qualitative framework for understanding the effect of crimping geometry, rather than a quantitative predictive model. Developing a quantitative micromechanical model based on this concept remains a topic for future research [17,24], as attempted in multi-strand models [17] and numerical modeling of entangled wire materials [24].

### 4.5. Synthesis and Broader Implications

The results demonstrate that the low-frequency dynamic performance of the WM-EPMM is influenced by its manufacturing parameters and depends on excitation amplitude. The increases in stiffness and dissipation with amplitude and density align with the general compaction behavior of porous friction-based materials [7,25,36]. The distinct frequency dependence observed within 1–5 Hz, where dissipation is constant but stiffness and loss factor shift, suggests that in this regime, the loading rate primarily affects elastic recovery and contact lag rather than frictional work per cycle. This observation may be relevant for applications where dominant disturbance frequencies are predictable and fall within this band, such as isolation systems for rotating machinery or mitigation of ground-borne vibrations.

The negative correlation of the crimped mesh mass ratio and crimping angle with stiffness, damping and loss factor presents a possible design parameter. By transitioning the dominant internal contact mode from line to point contact, and by introducing controlled structural compliance via inclined struts, the composite allows for the simultaneous reduction of the three metrics. However, it should be noted that this study employed a single-variable approach; potential interactions between parameters, such as density and mass ratio or crimping angle and amplitude, were not captured. Future studies using design of experiments or response surface methodology could quantify parameter interactions and enable more comprehensive optimization. Future work could focus on developing a quantitative micromechanical model [17,24], exploring performance under multi-axial loading, and extending dynamic characterization to higher frequencies [28,37] to establish a more complete frequency response map for the WM-EPMM. The fatigue behavior of the WM-EPMM under long-term cyclic loading also requires further investigation [29,30,38,39].

## 5. Conclusions

This study investigated the low-frequency dynamic mechanical properties of a Wire-Mesh Composite Elastic Porous Metallic Material (WM-EPMM). The main findings are as follows:

① The dynamic stiffness and energy dissipation capacity of the WM-EPMM increase with excitation amplitude and material density, while the loss factor shows a moderate decrease under these conditions.

② Within the tested low-frequency range of 1–5 Hz, the excitation frequency has a distinct influence: stiffness decreases slightly, the loss factor increases, and the dissipated energy per cycle remains largely unchanged, indicating an amplitude-dominated damping mechanism.

③ The mass ratio of crimped metal mesh (*C*) and its crimping angle (*α*) can be used to reduce dynamic stiffness, damping, and loss factor. Increasing either parameter leads to a decline in all three metrics.

④ The observed trends are interpreted through microstructural analysis. The transition from line contact in coils to point contact in mesh may contribute to the effect of the mass ratio, while the stability of equivalent inclined struts, conceptualized from the crimped geometry, provides a framework for understanding the influence of the crimping angle.

These results indicate that the low-frequency dynamic performance of the WM-EPMM can be adjusted by modifying its composition and architecture. The work provides experimental data and mechanistic insights that may assist in the development of metal rubber isolators for low-frequency vibration control applications. A limitation of this study is that each test condition was performed on a single specimen, and replicate testing was not feasible due to material and time constraints. Additionally, the current study was limited to loads up to 2.5 kN; the behavior under higher loads, where severe densification and potential plastic yielding of the wires may occur, remains to be clarified. Future work will include multiple specimens per condition to enable statistical analysis, investigating the mechanical response and failure mechanisms under higher loads, developing a quantitative micromechanical model, and extending the frequency range to fully characterize the dynamic response of the WM-EPMM.

## Figures and Tables

**Table 1:** Dynamic test matrix for WM-EPMM.

Varied Parameter	Levels
Excitation Amplitude (A)	1.5, 2.0, 2.5 mm
Excitation Frequency (f)	1, 3, 5 Hz
Density (*ρ*)	1.5, 1.75, 2.0, 2.25 g·cm^−3^
Mass Ratio (C)	0, 0.25, 0.5, 0.75, 1
Crimping Angle (α)	0°, 30°, 45°, 60°

**Table 2:** Dynamic properties under varying excitation amplitude (*ρ* = 2.0 g/cm^3^, *C* = 0.5, *α* = 45°, *f* = 1 Hz).

*A* (mm)	Δ*W* (J)	*η*	*k* (kN/mm)
1.5	0.574	0.167	0.401
2.0	0.927	0.158	0.452
2.5	1.391	0.150	0.556

Note: Each data point represents a single specimen. The load and displacement measurement accuracies of the testing machine were within ±1% and ±0.5%, respectively.

**Table 3:** Dynamic properties under varying excitation frequency (*ρ* = 2.0 g/cm^3^, *C* = 0.5, *α* = 45°, *A* = 2 mm).

*f* (Hz)	Δ*W* (J)	*η*	*k* (kN/mm)
1	0.927	0.158	0.452
3	0.922	0.168	0.434
5	0.928	0.207	0.393

**Table 4:** Dynamic properties under varying density (*C* = 0.5, *α* = 45°, *A* = 2 mm, *f* = 1 Hz).

*ρ* (g·cm^−3^)	Δ*W* (J)	*η*	*k* (kN/mm)
1.5	0.564	0.167	0.237
1.75	0.655	0.165	0.315
2.0	0.927	0.158	0.452
2.25	1.542	0.154	0.910

**Table 5:** Dynamic properties under varying crimping angle (*ρ* = 1.75 g·cm^−3^, *C* = 0.5, *A* = 2 mm, *f* = 1 Hz).

*α* (°)	Δ*W* (J)	*η*	*k* (kN/mm)
0	1.004	0.214	0.369
30	0.942	0.204	0.311
45	0.804	0.184	0.308
60	0.782	0.180	0.302

## Data Availability

The data presented in this study are available on request from the corresponding authors.
